# Healthcare worker perception of a global outbreak of novel coronavirus (COVID-19) and personal protective equipment: Survey of a pediatric tertiary-care hospital

**DOI:** 10.1017/ice.2020.415

**Published:** 2020-08-12

**Authors:** Pierre-Philippe Piché-Renaud, Helen E. Groves, Taito Kitano, Callum Arnold, Angela Thomas, Laurie Streitenberger, Laura Alexander, Shaun K. Morris, Michelle Science

**Affiliations:** 1 Division of Infectious Diseases, Hospital for Sick Children, Toronto, Canada; 2 Department of Paediatrics, University of Toronto, Toronto, Canada; 3 London School of Hygiene & Tropical Medicine, London, United Kingdom; 4 Occupational Health and Safety, Hospital for Sick Children, Toronto, Canada

## Abstract

**Objective:**

In this study, we aimed to capture perspectives of healthcare workers (HCWs) on coronavirus disease 2019 (COVID-19) and infection prevention and control (IPAC) measures implemented during the early phase of the COVID-19 pandemic.

**Design:**

A cross-sectional survey of HCWs.

**Participants:**

HCWs from the Hospital for Sick Children, Toronto, Canada.

**Intervention:**

A self-administered survey was distributed to HCWs. We analyzed factors influencing HCW knowledge and self-reported use of personal protective equipment (PPE), concerns about contracting COVID-19 and acceptance of the recommended IPAC precautions for COVID-19.

**Results:**

In total, 175 HCWs completed the survey between March 6 and March 10: 35 staff physicians (20%), 24 residents or fellows (14%), 72 nurses (41%), 14 respiratory therapists (8%), 14 administration staff (8%), and 14 other employees (8%). Most of the respondents were from the emergency department (n = 58, 33%) and the intensive care unit (n = 58, 33%). Only 86 respondents (50%) identified the correct donning order; only 60 (35%) identified the correct doffing order; but the majority (n = 113, 70%) indicated the need to wash their hands immediately prior to removal of their mask and eye protection. Also, 91 (54%) respondents felt comfortable with recommendations for droplet and/or contact precautions for routine care of patients with COVID-19. HCW occupation and concerns about contracting COVID-19 outside work were associated with nonacceptance of the recommendations (*P* = .016 and *P* = .036 respectively).

**Conclusion:**

As part of their pandemic response plans, healthcare institutions should have ongoing training for HCWs that focus on appropriate PPE doffing and discussions around modes of transmission of COVID-19.

The novel coronavirus disease (COVID-19) pandemic presents a significant infection control challenge within healthcare settings.^1,2^ Published studies from various countries have highlighted a significant proportion of healthcare-related infections as well as infections among healthcare workers (HCWs), especially in the early phase of the pandemic.^3–5^ These findings are consistent with healthcare-associated infections previously documented early within the Middle East respiratory syndrome coronavirus (MERS-CoV) outbreak and the severe acute respiratory syndrome (SARS) outbreak.^6–8^

A number of published studies from prior SARS and MERS-CoV outbreaks have highlighted the significant impact of such outbreaks on HCW morale and levels of concern that may impact perceptions and confidence in infection prevention and control (IPAC) measures as well as adherence to these approaches.^9–13^ Indeed, lack of confidence in institutional control measures can result in absenteeism, which in turn can have significant impacts on delivery of care within an outbreak setting.^13,14^ During the 2003 SARS outbreak in Canada, inconsistent use of PPE and lack of adequate infection control training were among the factors contributing to the infection of HCWs.^15^

In this study, we aimed to capture attitudes and knowledge of HCWs regarding COVID-19 and IPAC measures in the early phase of the COVID-19 pandemic, especially related to PPE. We also sought to identify factors influencing HCW knowledge and self-reported use of personal protective equipment (PPE), concerns about contracting COVID-19, and acceptance of the recommended IPAC precautions for COVID-19. This evaluation of the perspectives of HCWs on IPAC measures from the early phase of the pandemic provides invaluable information regarding the potential causes of initial nosocomial transmission of COVID-19 and ways to mitigate them moving forward.

## Methods

This is a cross-sectional study consisting of a self-administered survey for HCWs working at the Hospital for Sick Children, in Toronto, Canada. As the only pediatric tertiary-care hospital in Toronto, our center is uniquely positioned in regard to the current outbreak, given our previous experience with the SARS outbreak in 2003.^10^ The survey was distributed to clinicians and nonclinicians in emergency, intensive care, and pediatric wards as well as ambulatory clinics. Responses were recorded over a 5-day period from March 6 to March 10, 2020, using convenience sampling. An ethics review was completed through the quality improvement process at the hospital.

### Survey instrument

The survey instrument consisted of a series of questions developed by the Infectious Diseases, Occupational Health and Safety and IPAC unit at our hospital. The survey was distributed by email to an electronic mailing list of 951 clinical and nonclinical HCWs of the Hospital for Sick Children from the emergency department, intensive care unit, pediatric wards, and ambulatory clinics. An initial email was sent on March 6 with a reminder on March 9. Responses were collected anonymously using Research Electronic Data Capture (REDCap).^16^

The survey instrument was developed using previously published surveys delivered during similar viral outbreaks of global significance (SARS and MERS-CoV).^10–12,17–19^ Following initial validation by internal testing with IPAC and infectious diseases teams, the survey was subsequently pilot tested with a selected sample of HCWs to ensure comprehension and to resolve ambiguities. The finalized survey consisted of 19 questions divided in 3 sections: (1) baseline demographic characteristics and previous relevant training including PPE training, hand hygiene training, and COVID-19–specific PPE training; (2) knowledge, attitudes, and practices regarding PPE use; and (3) accessed sources of information and concerns regarding COVID-19. COVID-19 PPE training was done in person with a hands-on demonstration of donning and doffing by the nurse educators as well as by the Occupational Health and Safety team (ie, occupational hygienists). A video of the proper donning and doffing sequence was shown in addition to printed instructional materials and the Public Health Ontario donning and doffing posters. Information on the recommended equipment for care of patients with COVID-19 and other COVID-19 IPAC measures was also given. This training was made mandatory for all HCWs working at our institution, including new hires and current staff, starting in early January 2020. Our aim was to retrain as many HCWs as possible, but not all of them could be trained in-person for a number of reasons, including vacations and conflicting schedules.

To evaluate PPE knowledge, HCWs were asked the order in which they would don (put on) and doff (remove) PPE equipment. For both donning and doffing questions, a score of 1 was attributed if the correct order was identified, and a score of 0 was given for an incorrect order. The correct order for donning was defined in accordance with Public Health Ontario guidelines: (1) perform hand hygiene, (2) put on gown, (3) put on mask or N95 respirator, (4) put on eye protection, (5) put on gloves.^20^ The correct doffing order was defined as follows: (1) remove gloves, (2) remove gown, (3) perform hand hygiene, (4) remove eye protection, (5) remove mask or N95 respiratory, (6) perform hand hygiene.^20^ Respondents were also asked to report their usual use of PPE for droplet and/or contact precautions using a Likert scale: never (1), rarely (2), occasionally (3), frequently (4) and every time (5).

Because the current evidence suggests that the mode of transmission of SARS-CoV-2 is through direct contact and respiratory droplets, the Ontario Ministry of Health updated its recommendation on March 12 to the use of droplet and/or contact precautions for routine care of patients with COVID-19 and airborne precautions only for patients requiring aerosol-generating medical procedures (AGMPs).^21,22^ This was a change from the previous recommendation of N95 for all patients and based on experience from healthcare settings in which HCWs have not acquired COVID-19 while using droplet and contact precautions for routine care, including in other Canadian provinces.^23^ In anticipation of this change to be aligned with the provincial recommendations, the survey included questions around the acceptance of this recommendation, and what information would help HCWs feel comfortable making the change.

HCW concern regarding being exposed or contracting COVID-19 at work and outside work was assessed using the following 5-point Likert scale: not at all concerned (1), neutral (2), somewhat concerned (3), very concerned (4) and extremely concerned (5). Lastly, participants were prompted to provide comments on their use of PPE, IPAC precautions for COVID-19, and their satisfaction with the information provided to HCWs by the institution. The detailed survey can be found in Appendix 1 (online).

### Statistical analysis

Responses were analyzed using Statistical Package for Social Sciences version 25.0 (SPSS, Chicago, IL). Baseline demographic characteristics were reported for each category using absolute numbers and percentages. The χ^2^ test and the Fisher exact test were performed to estimate the significance among categorical study variables where appropriate. Analysis of variance (ANOVA) was performed to assess to estimate the significance between ordinal variables. Nonclinical HCWs (administration) were not included in the analysis of occupation on donning and doffing scores. Differences were considered statistically significant at *P* < .05. Missing answers were excluded from the analysis after confirmation that the underlying demographics were not substantially different from those analyzed, therefore minimizing selection bias. Thematic analysis was performed in respect to respondents’ free text comments to identify common themes.

## Results

### Characteristics of the study group

**Fig. 1.**
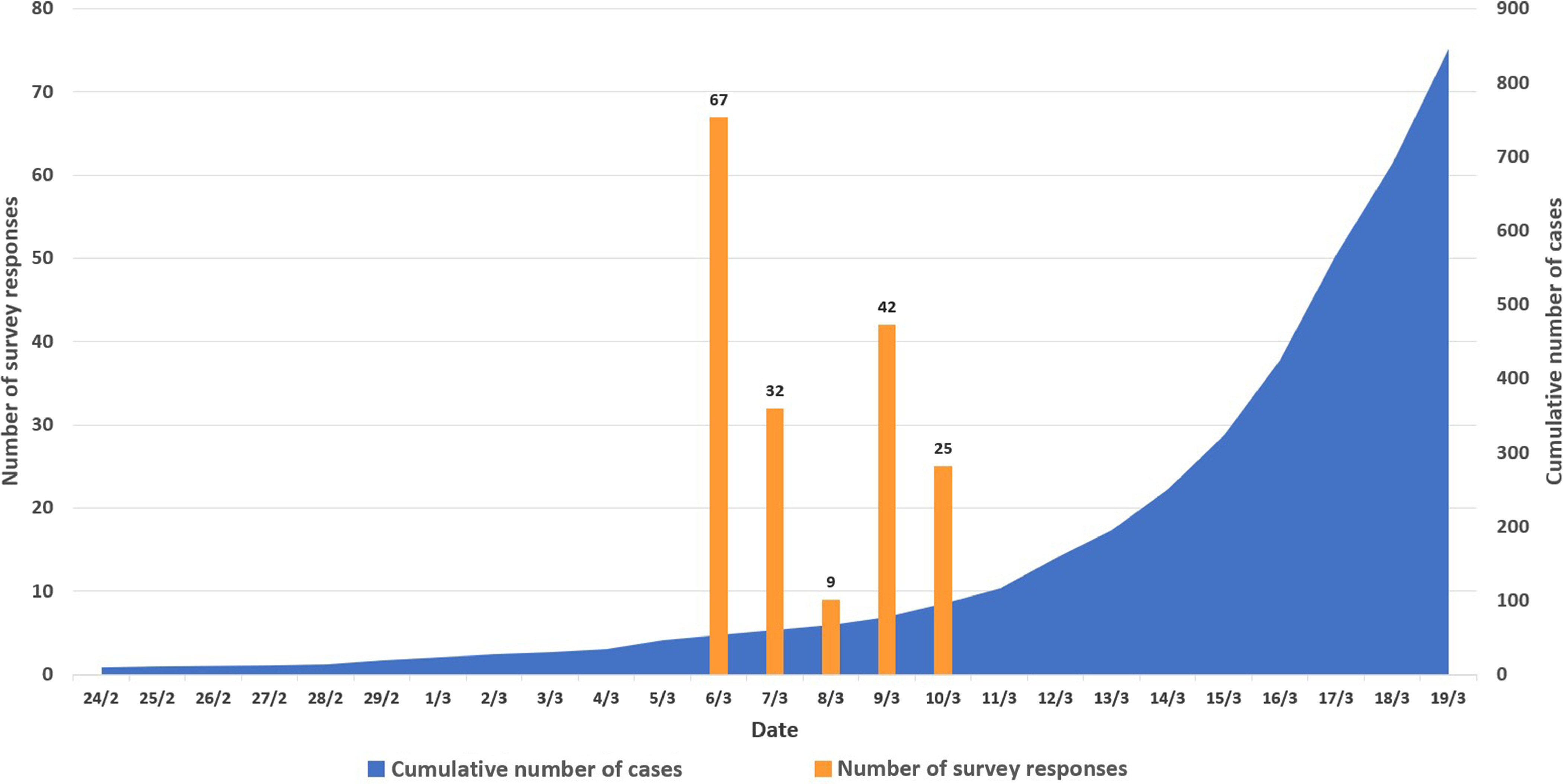
Timing of study in relation to the COVID-19 outbreak in Canada.

In total, 175 HCWs completed the survey, which corresponds to a response rate of 18.4%. Among them were 35 staff physicians (20%), 24 residents or fellows (14%), 72 nurses (41%), 14 respiratory therapists (8%), 14 administration staff (nonclinical, 8%), 14 other employees (8%), and 1 unknown. Also, 34 respondents (19%) reported having worked in the healthcare system during the 2003 SARS outbreak in Toronto. One-third of the respondents were from the emergency department (n = 58, 33%), one-third were from the intensive care unit (n = 58, 33%), and the other third were from the ward, the ambulatory clinic or other settings, such as specialty consulting services and patient support services. Detailed characteristics of the respondents are reported in Table 1. Survey responses were recorded in the 5 days immediately before the COVID-19 outbreak was declared a pandemic by the World Health Organization (WHO). The study timing and number of responses in relation to the COVID-19 outbreak in Canada and pandemic declaration are detailed in Figure 1. At the time of the survey, cases of COVID-19 in Canada were mainly reported among returning travelers or their contacts.

**Table 1.** Main Characteristics of Subjects With the Associated Donning and Doffing Scores

Characteristic		Donning			Doffing		
	No. (%)^a^	Donning Score	95% CI	*P* Value	Doffing Score	95% CI	*P* Value
Total mean score	172	0.50	0.43–0.58		.35	0.28–0.42	
**Received PPE training**	172			.23			**.003**
Yes	136 (79)	0.53	0.44–0.61		.40	0.32–0.49	
No	36 (21)	0.42	0.25–0.59		.14	0.02–0.26	
**Received hand hygiene training**	172			.14			**.007**
Yes	128 (74)	0.54	0.45–0.63		.41	0.32–0.49	
No	44 (26)	0.41	0.26–0.56		.18	0.06–0.30	
**Received COVID-19 PPE training**	163			.11			**<.001**
Yes	49 (30)	0.61	0.47–0.75		.55	0.41–0.70	
No	114 (70)	0.47	0.38–0.57		.26	0.18–0.35	
**Received all 3 trainings**	172			.08			**.001**
Yes	70 (41)	0.59	0.47–0.70		.50	0.38–0.62	
No	102 (59)	0.45	0.35–0.55		.25	0.16–0.33	
**Worked during SARS**	172			.28			.39
Yes	34 (20)	0.59	0.41–0.76		.41	0.24–0.59	
No	138 (80)	0.49	0.40–0.57		.33	0.25–0.41	
**Occupation (clinical)**	157			.29			.96
Staff physician	35 (22)	0.40	0.23–0.57		.34	0.18–0.51	
Resident or fellow	24 (15)	0.50	0.28–0.72		.38	0.17–0.58	
Nurse	71 (45)	0.56	0.45–0.68		.39	0.28–0.51	
Respiratory therapist	14 (9)	0.71	0.44–0.98		.43	0.13–0.73	
Other	13 (9)	0.62	0.31–0.92		.31	0.02–0.60	
Administration (nonclinical)	14						
**Department**	171			.66			.50
Emergency department	56 (33)	0.46	0.33–0.60		.30	0.18–0.43	
ICU	59 (34)	0.54	0.41–0.67		.44	0.31–0.57	
Ambulatory clinic	10 (6)	0.70	0.35–1.00^b^		.30	0^c^–0.65	
Inpatient units	22 (13)	0.45	0.23–0.68		.27	0.07–0.47	
Other	24 (14)	0.50	0.28–0.72		.33	0.13–0.54	
**Age, y**	166			.64			.93
<29	41 (25)	0.49	0.33–0.65		.37	0.21–0.52	
30–39	69 (41)	0.51	0.39–0.63		.33	0.22–0.45	
40–49	30 (18)	0.43	0.25–0.62		.40	0.21–0.59	
50–59	18 (11)	0.67	0.43–0.91		.28	0.05–0.51	
≥60	8 (5)	0.50	0.05–0.95		.38	0^a^–0.81	

Note. CI, confidence interval; PPE, personal protective equipment; SARS, severe acute respiratory syndrome; ICU, intensive care unit.

a Percentages for each demographic are reported as a fraction of the number of respondents who answered both of the analyzed variables.

b A score of 1.00 was given as the value obtained was above 1.

c A score of 0 was given as the value obtained was below 0.

### PPE knowledge and self-reported use

In total, 86 respondents (50%) identified the correct order for donning PPE, and 60 (35%) identified the exact correct doffing order. Also, 113 (70%) identified the need to perform hand hygiene prior to removal of their face mask and/or eye protection. Those who reported receiving previous training related to IPAC in the past 2 years (either general PPE training, hand washing training or COVID-19 specific PPE training) had significantly higher doffing PPE scores than those without reported training. Comparison of other baseline demographics and their impact on PPE knowledge are also presented in Table 1. No other factors had a statistically significant impact on PPE knowledge. With respect to usual PPE use for patients requiring droplet and/or contact precautions, respondents who received PPE training in the past 2 years reported using the most elements of PPE and more frequently than those who did not report PPE training. There was no statistical difference for the use of eye protection. These results are reported in Appendix 2 (online).

### Healthcare worker concerns regarding contracting COVID-19

In general, respondents were more concerned about being exposed or contracting COVID-19 at work than about contracting it outside work. Baseline demographics and other factors influencing concerns about contracting COVID-19 at work and outside work are detailed in Table 2. Notably, HCWs from the emergency department were the most concerned about contracting COVID-19 at work. Administration staff were the group most concerned about contracting COVID-19 outside work. Use of social media as a primary source of information was associated with increased concern of contracting COVID-19 both at work and outside work, whereas satisfaction with institution-provided information on COVID-19 was associated with lower concern. Every age group had similar concerns about contracting COVID-19 both at work and outside work. With respect to the use of droplet and/or contact precautions for the routine care of suspect or confirmed COVID-19 patients, 91 of 167 respondents (54%) felt comfortable with this recommendation. We detected a statistically significant association between HCW occupation and acceptance of the recommendations (P = .016). Nurses and respiratory therapists indicated that they would need more information compared with physicians, residents, and other staff.

**Table 2.** Factors Influencing HCW Concerns About Contracting COVID-19 at Work and Outside Work^a^

Variable		At Work			Outside Work		
	No. (%)	Concern	95% CI	*P* Value	Concern	95% CI	*P* Value
Total mean score	174	3.05	2.87–3.22		2.70	2.54–2.85	
**Age, y**	168			.76			.77
<29	42 (25)	3.00	2.64–3.36		2.55	2.25–2.85	
30–39	70 (42)	3.17	2.91–3.43		2.74	2.48–3.00	
40–49	30 (18)	2.87	2.40–3.33		2.80	2.42–3.18	
50–59	18 (11)	3.00	2.39–3.61		2.56	1.98–3.13	
≥60	8 (5)	3.25	2.38–4.12		2.87	2.05–3.70	
**Source of information used**							
Radio/Television	82 (47)	3.22	2.97–3.47	.06	2.84	2.62–3.06	.08
Not using	92 (53)	2.89	2.65–3.13		2.57	2.35–2.78	
Peers/colleagues	86 (49)	3.19	2.94–3.43	.11	2.77	2.55–2.98	.37
Not using	88 (51)	2.91	2.66–3.16		2.62	2.40–2.85	
Hospital website	110 (63)	3.06	2.85–3.28	.79	2.75	2.55–2.95	.33
Not using	64 (37)	3.02	2.73–3.31		2.59	2.34–2.84	
Social media	12 (74)	3.38	3.05–3.71	**.024**	2.98	2.67–3.28	**.034**
Not using	45 (26)	2.93	2.73–3.13		2.60	2.42–2.78	
MOH communication	80 (46)	3.24	2.97–3.51	**.043**	2.70	2.44–2.98	.96
Not using	94 (54)	2.88	2.66–3.11		2.69	2.50–2.88	
Public health website	65 (37)	3.28	3.01–3.55	**.041**	2.88	2.61–3.14	.075
Not using	109 (63)	2.91	2.69–3.13		2.59	2.40–2.78	
CDC website	95 (55)	3.11	2.88–3.33	.46	2.75	2.53–2.96	.47
Not using	79 (45)	2.97	2.70–3.25		2.63	2.40–2.86	
WHO website	106 (61)	3.12	2.91–3.33	.27	2.74	2.53–2.94	.52
Not using	68 (39)	2.93	2.63–3.23		2.63	2.39–2.88	
Journal articles	44 (25)	3.30	2.96–3.63	.097	2.84	2.52–3.16	.28
Not using	130 (75)	2.96	2.66–3.16		2.65	2.47–2.83	
**Satisfaction with the hospital website**	171			**<.001**			**.022**
Very satisfied	42 (25)	2.55	2.22–2.87		2.40	2.11–2.70	
Somewhat satisfied	62 (36)	3.29	3.01–3.57		2.79	2.53–3.06	
Neither satisfied nor dissatisfied	33 (19)	3.18	2.82–3.54		2.91	2.52–3.30	
Somewhat dissatisfied	6 (4)	4.33	3.79–4.88		3.50	2.40–4.60	
Very dissatisfied	4 (2)	4.50	3.58–5.00^a^		3.50	1.91–5.00^a^	
I have not consulted the hospital website	24 (14)	2.67	2.14–3.19		2.42	2.02–2.81	
**Worked during SARS**	174						
Yes	34 (20)	2.97	2.55–3.40	.67	2.62	2.54–2.89	.63
No	140 (80)	3.06	2.87–3.25		2.71	2.26–2.97	
**Occupation**	172			.059			**.007**
Staff physician	34 (20)	3.38	3.03–3.74		2.91	2.56–3.26	
Resident or fellow	24 (14)	2.75	2.35–3.15		2.58	2.14–3.03	
Nurse	72 (42)	2.93	2.64–3.22		2.40	2.16–2.64	
Respiratory therapist	14 (8)	3.43	2.94–3.92		2.79	2.32–3.25	
Administration	14 (8)	3.43	2.65–4.20		3.43	2.72–4.13	
Other	14 (8)	2.57	1.83–3.31		3.00	2.49–3.51	
**Department**	173			**.049**			.23
Emergency department	57 (33)	3.39	3.06–3.71		2.65	2.33–2.96	
ICU	59 (34)	2.78	2.50–3.06		2.56	2.32–2.80	
Ambulatory clinic	10 (6)	3.10	2.39–3.81		3.00	2.33–3.67	
Inpatient units	22 (13)	2.77	2.34–3.20		2.59	2.21–2.97	
Other	25 (14)	3.12	2.61–3.63		3.08	2.64–3.52	
**Received PPE training**	174			.91			.062
Yes	136 (78)	3.05	2.86–3.24		2.62	2.45–2.78	
No	38 (22)	3.03	2.61–3.44		2.97	2.66–3.38	

Note. CI, confidence interval; HCW, healthcare worker; MOH, Ministry of Health; WHO, World Health Organization; CDC, Centers for Disease Control and Prevention; SARS, severe acute respiratory syndrome; PPE, personal protective equaipment.

a Scoring system: 1: not at all concerned; 2: neutral; 3: somewhat concerned; 4: moderately concerned; 5: extremely concerned.

b A score of 5.00 was given if the value obtained was >5.

### Facilitators identified through respondents’ comments

Thematic analysis of the respondents’ comments allowed us to identify facilitators for PPE implementation, acceptance of COVID-19 IPAC measures, and information transmission regarding COVID-19. HCWs indicated that they would be more likely to accept the recommendation for droplet and/or contact precautions for the routine care of patients with COVID-19 if they were more confident in their knowledge of PPE donning and doffing. They also had concerns about PPE availability in their workplace and feared that an impending shortage could influence guidance around IPAC measures. Respondents reported that thorough information on transmission modes of COVID-19 would facilitate their acceptance of the recommendation. Respondents preferred information that was tailored to their occupation and provided by the fewest sources possible.

## Discussion

Our findings provide insight into HCW attitudes and knowledge of COVID-19 and the related IPAC measures during the early phase of the pandemic. COVID-19–specific PPE training had the most significant impact on HCWs knowledge of PPE donning and doffing. The early implementation of IPAC and PPE trainings may therefore have mitigated the nosocomial spread of COVID-19. HCWs were most concerned about being exposed or contracting COVID-19 at work, and half of the respondents from our study reported being comfortable with recommendations for droplet and/or contact precautions for routine care of patients with COVID-19.

Approximately one-third of the respondents were able to correctly identify the appropriate order to remove PPE equipment. This finding was of concern because incorrect doffing order has been shown to lead to increased contamination of HCW clothing and the surrounding environment, potentially leading to HCW infections.^24,25^ PPE training with a focus on PPE doffing was identified as a priority for all HCWs caring for patients with suspected or confirmed COVID-19, regardless of their previous work experience. Given that 30% of HCWs did not report the need to perform hand hygiene immediately before removal of face mask and/or eye protection in our survey, we identified this as an important focus of PPE training at our institution because it was a source of HCW contamination during the SARS outbreak.^26^ With the feedback from this survey, we also created an online learning module for all HCWs at our institution that incorporated lessons learned, including modes of transmission of COVID-19, proper protection needed for specific clinical tasks, and a focus on the importance of the correct sequence of doffing PPE. The online module made it easier to reach all HCWs and to provide further reinforcement and learning opportunities, compared to in-person trainings.

Notably, our study captured HCW concerns about contracting COVID-19 early in the outbreak, just days before it was declared a pandemic by the WHO, at which time not all HCWs had received PPE refresher training. Having a thorough insight into HCW attitudes and knowledge of IPAC measures from the early phase of the pandemic is important to understanding the causes of COVID-19 infection among HCWs. Most HCW infections occurred early in the COVID-19 outbreak.^5,27^ In Ontario, 4,230 HCWs have been infected, which represents 17.5% of the 24,202 confirmed COVID-19 cases as of May 14, 2020. As few as 3.1% of the infected HCWs were documented to have acquired COVID-19 nosocomially.^27^ Unfortunately, no data on the adequacy of PPE used by HCWs infected nosocomially are available. Based on the results of our study, initial gaps in HCW PPE knowledge, especially related to doffing order, may have contributed to nosocomial infections among HCWs in the early phase of the pandemic.

In our study, HCWs from the emergency department had the highest level of concern regarding contracting COVID-19 at work, which is not surprising given the volume and acuity of patients they see. This finding is in keeping with previous experience of the 2003 SARS outbreak in Toronto, during which hospital emergency departments were important sites for SARS transmission in the early part of the epidemic.^28^ Recently, Tan et al^29^ assessed the psychological impacts of COVID-19 on HCWs in Singapore, and 68 of the 470 surveyed participants (14.5%) screened positive for anxiety. In our study, using social media as a source of information was strongly associated with HCW concerns regarding contracting COVID-19, both at work and outside work. This finding affirms previous assumptions that the use of social media may induce anxiety regarding COVID-19 in users and therefore should not be promoted as the main source of information.^30^ However, it is important to acknowledge the possibility that direction of causality in our study may be the reverse, and HCWs that have greater concerns about contracting COVID-19 are more likely to consume more information surrounding the pandemic, including a greater diversity of information sources. This hypothesis is reinforced by the fact that using public health website and MOH communications as sources of information was also associated with increased concerns about contracting COVID-19 at work.

Our study has some limitations. First, respondents were recruited using convenience sampling, which could therefore limit the external validity of our study. The studied population was relatively young: 42% were aged 30–39 years. Although most of our results reflect those of previous studies on viral outbreaks of global significance, a lack of standardized methodologies between studies limits such comparison. Moreover, in view of the cross-sectional nature of the study, we were only able to capture HCW knowledge and perceptions within a limited period.

This study has provided important insight into HCW knowledge and attitudes toward COVID-19 and IPAC measures during the early phase of the pandemic. To ensure that IPAC responses accurately reflect gaps in knowledge and to identify specific facilitators to continuous improvement, follow-up assessments are also required. A consistent framework through which IPAC knowledge can be assessed should also be developed, allowing for comparisons at national and international levels as well as rapid dissemination of hospital epidemic response plans. With this survey, we aimed to contribute to this important topic and to provide an adaptable framework with which to generate context-specific IPAC plans.

## Supplementary material

For supplementary material accompanying this paper visit http://dx.doi.org/10.1017/ice.2020.415. click here to view supplementary material
