# Supplementary material for: Healthcare worker perception of a global outbreak of novel coronavirus (COVID-19) and personal protective equipment: Survey of a pediatric tertiary-care hospital
Source: Infect Control Hosp Epidemiol. 2020 Aug 12:1–7. doi: 10.1017/ice.2020.415 (PMC7468688; doi:10.1017/ice.2020.415)
Supplement: Supplementary file 1 [file S0899823X20004158sup001.docx]

**Appendix 1: COVID-19 Staff Survey**

Dear Colleague,

In January 2020, a novel coronavirus (COVID-19) was identified as the cause of an outbreak of pneumonia originating in China. The situation is rapidly evolving with cases identified in several countries and hospitals across Ontario are planning for a possible pandemic. With this in mind, we are conducting a hospital-wide survey of knowledge, attitudes and infection prevention and control practices of hospital staff members.

We aim to use the information collected from this survey to identify if there are knowledge and/or practice gaps with respect to COVID-19 and the use of infection prevention and control practices. Using this information, we hope to improve existing resources for our staff members.

Your completion of this survey is voluntary and at any point you can discontinue survey completion. You are free to leave blank any questions that you do not wish to answer. This survey is completely anonymous and should take less than 5 minutes to complete. Please note that some questions will refer to the 2003 SARS outbreak.

We greatly appreciate your time and effort.

*General information* (please check all that apply)

1. **Age:** less than 29  30 – 39  40 – 49  50 - 59  60 or over  Prefer not to say
2. **Occupation:** Staff physician  Resident or fellow  Nurse  Student   Respiratory Therapist  Patient Support Staff  Administration  Anesthesia  Physiotherapist  Occupational Therapist  Other: specify _______________
3. **In which department are you *primarily* based?**

Emergency Department  Inpatient Unit  Intensive care unit  Anesthesia  Ambulatory clinic

Other: please specify __________________________________________

1. **How many years have you worked at SickKids?**

Less than 5   5 – 10  11 – 15  16 – 20  Over 20 years 

1. **Were you working in healthcare in Ontario during the 2003 SARS outbreak?** YES  NO

**If answered yes, the following question will appear:**

**6.a) Did you have any direct experience taking care of a patient with SARS?** YES  NO 

1. **Did you, a colleague or a close family member become exposed or infected during the SARS outbreak?**

YES  NO  PREFER NOT TO SAY

*Personal Protective Equipment*

1. **Have you received training or orientation about the following infection prevention and control (IPAC) measures within? (Check all that apply)**
   - Personal Protective Equipment (PPE) donning and doffing
   - Hand hygiene
   - Other
     - - Please specify ________________
   - No training on these measures
2. **What is the recommended order for donning Personal Protective Equipment (PPE) when encountering patients or their environment needing enhanced Droplet/Contact precautions? (Please number 1 to 5)**
   - Put on mask or N95 respirator ( )
   - Put on gown ( )
   - Put on gloves ( )
   - Perform hand hygiene ( )
   - Put on eye protection ( )

1. **What is the recommended order for removing Personal Protective Equipment (PPE) when encountering patients or their environment needing enhanced Droplet/Contact precautions? (Please number 1 to 6)**
   - Perform hand hygiene ( )
   - Remove mask or N95 respirator ( )
   - Remove gown ( )
   - Remove gloves ( )
   - Perform hand hygiene ( )
   - Remove eye protection ( )
2. **In general, how frequently do you use the following PPE when encountering patients or their environment requiring Droplet/Contact precautions?**

- Every time
- Frequently (More than 75% of the time)
- Occasionally (25-75% of the time)
- Rarely (Less than 25% of the time)
- Never

1. **Have you participated in any PPE training specific for managing patients with suspected or confirmed COVID-19?**

YES   NO  NOT SURE

1. **At SickKids, what PPE is currently recommended for routine care (not aerosol-generating procedures) when encountering patients or their environment with suspected or confirmed COVID-19 infection? Please check all that apply.**

- Wearing a surgical mask
- Wearing a N95 respirator
- Hand washing
- Use of gown and gloves
- Wearing eye protection/face shield  
  - Wearing protective suit/hood

1. **At SickKids, what PPE is currently recommended for aerosol-generating procedures when encountering patients or their environment with suspected or confirmed COVID-19 infection? Please check all that apply.**

- Wearing a surgical mask
- Wearing a N95 respirator
- Hand washing
- Use of gown and gloves
- Wearing eye protection/face shield  
  - Wearing protective suit/hood

1. **For patients with suspected or confirmed COVID-19, current guidance from the Ontario Ministry of Health recommends use of N95 respirators as part of PPE for all care interactions. If guidance were to change to recommend standard Droplet/Contact precautions for routine care (ie. N95 respirator only during aerosol generating procedures), would you feel comfortable with this practice change?**

- I already feel comfortable
- I would need additional information
  - Please specify: ___________________________

*COVID-19 Information*

1. **Please indicate what resources you use to stay up to date on the latest information for the COVID-19 outbreak and what resource do you find the most reliable?**

Currently using Most reliable (check one)

- Radio/television
- Peers/colleagues
- SickKids website
- Social media (ie. *Twitter, Facebook,* etc)
- Ministry of Health communications
- Public Heath Ontario website
- Journal articles
- CDC website
- I don’t keep up to date
- Other

Please specify : ­­­­­­­­­­­­­­­­­­_______________________

1. **Are you concerned about being exposed or infected by COVID-19?**

At work Outside of work

- Extremely concerned
- Moderately concerned
- Somewhat concerned
- Neutral
- Not at all concerned

1. **Do you use a mask in public spaces outside of SickKids?** YES  NO
2. **Are you satisfied by the information provided on the SickKids’ website on how the COVID-19 outbreak impacts you at SickKids?**

- Very satisfied
- Somewhat satisfied
- Neither satisfied nor dissatisfied
- Somewhat dissatisfied
- Very dissatisfied
- I have not consulted the information on the website

1. **We invite your ideas about how information sharing and learning about COVID-19 could be improved at SickKids:**

## Appendix 2: Impact of PPE training on the self-reported use of the elements of PPE for droplet/contact precautions

|  | **n (%)** | **Never (1)** | **Rarely (2)** | **Occasionally (3)** | **Frequently (4)** | **Every time (5)** | **Mean score** | **CI (95%)** | **p-value** |
| --- | --- | --- | --- | --- | --- | --- | --- | --- | --- |
| **Mask, n (%)** | 170 | 5 (3) | 6 (4) | 16 (9) | 41 (24) | 102 (60) | 4.35 | 4.20 - 4.50 | **0.037** |
| PPE training | 136 (80) | 2 (1) | 3 (2) | 13 (10) | 35 (26) | 83 (61) | 4.43 | 4.28 - 4.57 |  |
| No PPE training | 34 (20) | 3 (9) | 3 (9) | 3 (9) | 6 (18) | 19 (56) | 4.03 | 3.56 - 4.50 |  |
| **Gown, n (%)** | 167 | 6 (4) | 24 (14) | 26 (15) | 46 (28) | 65 (39) | 3.84 | 3.66 - 4.02 | **0.029** |
| PPE training | 133 (80) | 1 (1) | 19 (14) | 18 (14) | 44 (33) | 51 (38) | 3.94 | 3.75 - 4.12 |  |
| No PPE training | 34 (20) | 5 (15) | 5 (15) | 8 (24) | 2 (6) | 14 (41) | 3.44 | 2.91 - 3.97 |  |
| **Gloves, n (%)** | 167 | 4 (2) | 5 (3) | 18 (11) | 50 (30) | 90 (54) | 4.30 | 4.15 - 4.44 | **0.001** |
| PPE training | 133 (80) | 0 (0) | 3 (2) | 12 (9) | 44 (33) | 74 (56) | 4.42 | 4.29 - 4.55 |  |
| No PPE training | 34 (20) | 4 (12) | 2 (6) | 6 (18) | 6 (18) | 16 (47) | 3.82 | 3.33 - 4.31 |  |
| **Eye protection, n (%)** | 163 | 25 (15) | 66 (40) | 37 (23) | 11 (7) | 24 (15) | 2.65 | 2.46 - 2.84 | 0.55 |
| PPE training | 129 (79) | 14 (11) | 58 (45) | 34 (26) | 9 (7) | 14 (11) | 2.62 | 2.43 - 2.82 |  |
| No PPE training | 34 (21) | 11 (32) | 8 (24) | 3 (9) | 2 (6) | 10 (29) | 3.24 | 2.18 - 3.35 |  |

**NB.** All percentages reported for each response are calculated based on the “n” from each row
